# 基于共价有机骨架材料的磁固相萃取-超高效液相色谱-串联质谱法测定水中8种磺胺类抗生素

**DOI:** 10.3724/SP.J.1123.2024.12002

**Published:** 2025-08-08

**Authors:** Guangnian YUAN, Jiping MA, Yuankun LI, Shuang LI

**Affiliations:** 青岛理工大学环境与市政工程学院，山东 青岛 266520; School of Environmental and Municipal Engineering，Qingdao University of Technology，Qingdao 266520，China

**Keywords:** 共价有机骨架材料, 磁固相萃取, 超高效液相色谱-串联质谱, 磺胺类抗生素, 环境水体, covalent organic framework materials, magnetic solid-phase extraction（MSPE）, ultra performance liquid chromatography-tandem mass spectrometry（UPLC-MS/MS）, sulfonamide antibiotics（SAs）, environmental water

## Abstract

磺胺类抗生素（SAs）可被人体或动物代谢，随后通过地表径流进入环境，对生态环境和人类健康造成危害。因此，开发一种针对水中SAs的简单、快速、高效且灵敏的分析方法至关重要。本文采用原位合成法制备了磁性共价有机骨架材料Fe_3_O_4_@TpDT，并将其作为磁固相萃取吸附剂，用于环境水体中8种SAs的吸附与富集。为获得最佳萃取效率，本研究系统考察了萃取条件（材料用量、水样pH、吸附时间）和洗脱条件（洗脱溶剂的类型及体积、洗脱时间）。优化结果显示，Fe_3_O_4_@TpDT吸附剂在6 min内即可完全吸附目标化合物，使用1 mL甲醇洗脱2 min，目标化合物便可充分解吸。在最佳萃取条件下，结合超高效液相色谱-串联质谱（UPLC-MS/MS）技术，本研究建立了一种测定环境水样中8种SAs的新方法。方法学验证结果表明，8种SAs在各自的线性范围内呈良好的线性关系（相关系数（*r*^2^）≥0.992 6），检出限（LOD）和定量限（LOQ）分别为0.80~3.44 ng/L和2.66~11.47 ng/L。在空白水样（水厂水）中进行３个水平（50、500和800 ng/L）的加标回收试验，8种SAs的回收率为73.0%~112.9%，日内和日间精密度分别为4.4%~12.5%和8.7%~19.2%。最后将该方法应用于水厂水、水库水和海水中SAs的测定，结果表明，水厂水均未检出8种SAs，在水库水中检出3种SAs，在海水中检出1种SAs；在３个加标水平（50、500和800 ng/L）下，水库水和海水中8种SAs的回收率为55.0%~100.9%，相对标准偏差（RSD）为1.3%~14.0%。该方法操作简单，萃取时间短且具有良好的准确度和精密度，为环境水体中SAs的富集检测提供了技术支撑。

## Abstract (en)

Sulfonamide antibiotics（SAs） are artificially synthesized antibiotics， which have been widely used in the medical industry and animal husbandry because of their broad antibacterial property. Unfortunately， SAs can be metabolized by humans or animals and enter the environment through surface runoff， posing threats to environmental ecology and human health. Therefore， it is crucial to develop simple， rapid， highly efficient， and sensitive analytical methods for detecting SAs in water. Sample pretreatment is important for the extraction and enrichment of pollutants from environmental water. Magnetic solid-phase extraction（MSPE） is a new sample pretreatment method， which uses magnetic materials as adsorbents dispersed in solution， and rapid separation can be achieved by the aid of external magnets. Because of its advantages of short enrichment time， less organic solvent consumption， and easy separation of adsorbents， MSPE has attracted much attention. The key to MSPE is the preparation of highly selective magnetic adsorbents. Covalent organic frameworks（COFs） have the advantages of large surface， chemical and thermal stability， tunable porous structure， low density and easy functionalization. Therefore， a sample pretreatment method combining MSPE and COF for the analysis of SAs in complex matrices is very promising. The contents of SAs in environmental water are low， ultra performance liquid chromatography-tandem mass spectrometry（UPLC-MS/MS） has high sensitivity and accuracy. In this paper， the magnetic COF material Fe_3_O_4_@TpDT was prepared by in-situ synthesis method， which was used as an MSPE adsorbent for the adsorption and enrichment of eight SAs in environmental waters. Based on the *π-π* conjugation， hydrogen bonding， electrostatic interaction and molecular size effect between Fe_3_O_4_@TpDT and SAs， in combination with UPLC-MS/MS， this study established a new method for the determination of eight SAs in environmental water samples. The successful synthesis of Fe_3_O_4_@TpDT was confirmed using scanning electron microscopy（SEM）， Fourier transform infrared spectroscopy（FT-IR）， X-ray diffraction（XRD）， surface area analyzer and X-ray photoelectron spectroscopy（XPS） spectrum. To obtain the optimal extraction efficiency， the extraction conditions（dosage of material， pH of water sample， extraction time） and elution conditions（type and volume of elution solvent， elution time） were systematically investigated. The optimization results show that the Fe_3_O_4_@TpDT adsorbent can completely adsorb the target compounds within 6 min. After elution with 1 mL of methanol for 2 min， the target compounds can be fully desorbed. The target compound were separated on an ACQUITY UPLC BEH C18 column（100 mm×2.1 mm， 1.7 μm） and gradient elution was carried out with methanol-0.1% formic acid solution as the mobile phase. Multiple reaction monitoring（MRM） was conducted in the positive electrospray ionization mode and the ion source temperature and ion source voltage were set to 500 ℃ and 5 kV， respectively. Under the optimal extraction conditions， the enrichment factors（EFs） of Fe_3_O_4_@TpDT for eight SAs were 39‒48. The methodological verification results indicated that the eight SAs had good linear relationships（correlation coefficients（*r*^2^）≥0.992 6）， and the limits of detection（LODs） and quantification（LOQs） were 0.80‒3.44 ng/L and 2.66‒11.47 ng/L， respectively. The spiked recovery tests were conducted at three spiked levels（50， 500 and 800 ng/L） in blank water samples（tap water plant effluent）. The recoveries of the eight SAs were 73.0%‒112.9%， and the intra- and inter-day precisions were 4.4%‒12.5% and 8.7%‒19.2%， respectively. Finally， this method was successfully applied to the determination of eight SAs in environmental water samples（tap water plant effluent， reservoir water and seawater）. The results showed that no SAs were detected in tap water plant effluent， three SAs were detected in the reservoir water， and one SA was detected in the seawater. At three spiked levels（50， 500 and 800 ng/L）， the recoveries of eight SAs in reservoir water and seawater were 55.0%‒100.9%， and the relative standard deviations（RSDs） were 1.3%‒14.0%. This method is simple to operate， has a short extraction time， and has good accuracy and precision， providing technical support for the enrichment detection of SAs in environmental water samples.

磺胺类抗生素（sulfonamide antibiotics， SAs）是一类具有对氨基苯磺酰胺结构的药物的总称，作为抗菌药物，其在人类医学和兽医学领域得到了广泛应用^［1］^。由于SAs能够通过人体或动物代谢进入环境，并且具有一定的光稳定性和热稳定性，因此目前SAs在土壤^［2］^、地下水^［3］^、地表水^［4］^、沉积物^［5］^等环境中均以痕量水平存在。研究表明，SAs引发的抗菌性可能会显著降低微生物的活性^［6］^；同时，SAs会在食物链中积累，进而导致毒性效应局部增强^［7］^；此外，SAs进入环境还会诱发抗生素耐药性基因的产生，对环境生态和人类健康构成威胁。因此，亟需掌握SAs在环境中的浓度分布情况及其环境效应，其中，对环境中SAs分析检测方法的研究具有重要意义。

现阶段，SAs的检测方法主要包括液相色谱-串联质谱法（LC-MS/MS）^［8］^、高效液相色谱法（HPLC）^［9］^、毛细管电泳法^［10］^以及酶联免疫法^［11］^等。其中，LC-MS/MS凭借其特异性强、灵敏度高等优势，常被应用于水中抗生素的分析检测工作。由于SAs在环境水样中的含量较低，通常处于ng/L级水平，并且水样中的基质会对SAs的检测产生干扰，所以在利用仪器进行分析之前，需采用样品前处理技术对水样进行处理^［12］^。环境水体中SAs的前处理方法有固相萃取（SPE）^［13］^、分散固相萃取（DSPE）^［14］^、固相微萃取（SPME）^［15］^、分散液液微萃取^［16］^和磁固相萃取（MSPE）法^［17］^等。与传统的SPE技术相比，MSPE具有操作简单、耗时短、有机溶剂消耗量少等优点^［18］^。例如，我们课题组^［19］^制备了一种磁性共价有机骨架（covalent organic framework， COF）材料Fe_3_O_4_@TpBD，并结合超高效液相色谱-串联质谱（UPLC-MS/MS）技术，建立了一种针对水中11种芳香类消毒副产物（DBPs）的分析方法；运用该方法进行样品前处理时，仅需7 min。Wang等^［20］^采用碳化后的Fe_3_O_4_作为载体，制备了一种磁性金属有机骨架材料Fe_3_O_4_@C@UiO-66，该材料可有效萃取环境水和果汁中的手性农药。

COFs是一类多孔有机晶体材料，它具备比表面积大、孔隙率高、热稳定性好等特性，同时还拥有可调节的孔径以及可进行表面功能化的优点^［21］^。由于COFs材料具有独特的孔隙结构和丰富的吸附位点，能够高效富集环境中的目标分析物，因此它已被广泛应用于样品前处理领域^［22，23］^。基于上述COFs材料的优点，中山大学欧阳钢锋团队^［24］^以苯-1，3，5-三乙醛和1，3，5-三（4-氨基苯基）苯为配体，在不锈钢丝表面成功合成了COFs材料。该材料对菊酯杀虫剂、有机磷和有机氯类化合物具有高效富集能力。济南大学孙敏团队^［25］^通过将SiO_2_@COF进行碳化处理，合成了SiO_2_@C-COF材料；将该材料与SPE技术结合，再利用高效液相色谱-二极管阵列检测器（HPLC-DAD），建立了可用于在线分析水中四溴双酚A衍生物和邻苯二甲酸酯的方法。我们课题组^［26］^以2，4，6-三甲酰基间苯三酚和联苯胺为配体合成了COFs材料，并将其负载到尼龙膜上，实现了对环境水体中六溴环十二烷的吸附富集。

本研究以三醛基间苯三酚（TP）和4，4-二氨基三联苯（DT）为配体，通过席夫碱反应合成TpDT COF材料，再利用原位合成法将TpDT COF与Fe_3_O_4_结合，制备了一种磁性COF材料Fe_3_O_4_@TpDT。将该材料作为MSPE吸附剂，结合UPLC-MS/MS技术，建立了一种可快速富集并灵敏检测环境水样中8种SAs的分析方法，以期为环境水样中SAs的残留分析提供技术支持。

## 1 实验部分

### 1.1 仪器、试剂与材料

QTRAP 3500超高效液相色谱-三重四极杆质谱仪（美国AB Sciex公司），Frontier傅里叶变换红外光谱仪（美国PerkinElmer公司），Sigma300电子扫描显微镜（德国Zeiss公司），D8 Advance型X-射线衍射仪（美国Bruker公司），ESCALAB 250Xi型X射线光电子能谱（美国Thermo Fisher公司），ASAP2460型氮气吸附-脱附比表面积分析仪（美国Micromeritics公司），Millipore D-24UV超纯水机（美国Millipore公司），涡旋混匀仪（上海沪析有限公司），HH-G1型数显恒温水浴锅（常州普天仪器制造有限公司），KQ5200DE型数控超声波清洗器（昆山市超声仪器有限公司），DZF-6020型真空干燥箱（上海一恒科学仪器有限公司）。

8种SAs标准品：磺胺甲基嘧啶（SMI，纯度≥99%）和磺胺异噁唑（SIZ，纯度≥98%）购自上海阿拉丁生化科技股份有限公司，磺胺甲噁唑（SMX，纯度≥98%）和磺胺甲氧哒嗪（SMP，纯度≥99%）购自上海麦克林生化科技股份有限公司，磺胺间甲氧嘧啶（SMM，纯度≥98%）和苯甲酰磺胺（SB，纯度≥98%）购自上海吉至生化科技有限公司，磺胺二甲基嘧啶（SMZ，纯度≥98%）和磺胺氯哒嗪（SCP，纯度≥99%）购自上海安谱实验科技股份有限公司，8种SAs的化学结构和酸碱系数见附图1（www.chrom-China.com）。

Fe_3_O_4_纳米粒子（粒径200 nm）购自上海麦克林生化科技股份有限公司，TP（纯度97%）购自吉林中科研伸科技有限公司，DT（纯度99%）购自上海迈瑞尔生化科技有限公司，甲醇（分析纯）和四氢呋喃（THF，分析纯）购自天津市北辰方正试剂厂，乙二胺四乙酸二钠（Na₂EDTA，分析纯）购自国药集团化学试剂有限公司。

实验所用水库水和海水采集自青岛市丁家河水库和金沙滩，水厂水采集自青岛市某自来水厂。采集后的水样经0.45 μm玻璃纤维滤膜过滤后，储存于棕色玻璃瓶中，在4 ℃下保存备用。

### 1.2 标准溶液的配制

分别准确称量10 mg的8种SAs标准品（精确至0.1 mg），用甲醇溶解并定容至10 mL，配制成质量浓度为1 000 mg/L的SAs单标储备液，于‒18 ℃下避光保存。移取100 μL各单标储备液，用甲醇混合稀释并定容至10 mL，配制成质量浓度为10 mg/L的混合标准工作溶液，将其置于棕色容量瓶中，于‒18 ℃下避光保存。临用前用甲醇逐级稀释至所需质量浓度。

### 1.3 材料制备

将30 mg 粒径为200 nm的Fe_3_O_4_纳米粒子和20 mg DT溶解于20 mL THF中，超声处理30 min，然后机械搅拌30 min。之后，将4 mL含有16 mg TP的THF溶液逐滴缓慢加入到上述混合液中，并在50 ℃下进行加热回流，同时机械搅拌3 h。反应结束后，用磁铁收集生成的颗粒，并分别用THF和甲醇各洗涤3次。最后，将产物置于45 ℃的烘箱中干燥，即可得到Fe_3_O_4_@TpDT材料。

### 1.4 样品前处理

称取25 mg Fe_3_O_4_@TpDT磁性材料，置于50 mL离心管中，向离心管内加入50 mL水样，再加入10 mg Na₂EDTA，用1 mol/L盐酸调节溶液pH至约4，涡旋振荡6 min后，将磁铁置于离心管外壁，通过磁性作用分离出固体物质并弃去上清液；随后，向离心管中加入1 mL甲醇，涡旋洗脱2 min，再用磁铁分离出洗脱液；将洗脱液过0.22 μm有机滤膜后，待上样进行UPLC-MS/MS分析。

### 1.5 仪器条件

色谱条件：ACQUITY UPLC BEH C18色谱柱（100 mm×2.1 mm， 1.7 μm），柱温：30 ℃，进样量：10 µL。流动相：0.1%甲酸水溶液（A）和甲醇（B），流速：0.3 mL/min。梯度洗脱程序：0~0.6 min，65%A；0.6~1.0 min，65%A~10%A；1.0~8.0 min，10%A；8.0~8.1 min，10%A~90%A；8.1~10.0 min，90%A。

质谱条件：ESI源，正离子模式；多反应监测模式；离子源温度：500 ℃；离子源电压：5 kV；气帘气压力：3.5×10^5^ Pa；雾化气压力：5×10^5^ Pa；辅助器压力：6.0×10^5^ Pa。8种SAs的保留时间和其他质谱参数见表1。

**表1:** 8种SAs的保留时间和质谱参数

Analyte	*t_R_* /min	Precursor ion（*m/z*）	Product ions（*m/z*）	DP/V	CEs/eV
Sulfamerazine（SMI）	2.53	265.1	156.0^*^， 171.9	74	25， 24
Sulfadimidine（SMZ）	2.80	279.1	185.9^*^， 155.9	70	24， 24
Sulfamethoxypyridazine（SMP）	2.92	280.8	156.1^*^， 126.1	70	24， 26
Sulfachloropyridazine（SCP）	3.15	285.0	156.1^*^， 126.1	70	24， 26
Sulfamethoxazole（SMX）	3.23	254.1	108.2^*^， 155.6	80	23， 38
Sulfamonomethoxine（SMM）	3.35	281.1	156.1^*^， 214.6	74	23， 23
Sulfisoxazole（SIZ）	3.42	267.8	155.9^*^， 113.0	65	19， 22
Sulfabenzamide（SB）	3.68	277.0	155.9^*^， 108.0	63	19， 34

DP： declustering potential； CEs： collision energies； * quantitative ion.

## 2 结果与讨论

### 2.1 材料表征

利用SEM对Fe_3_O_4_@TpDT磁性材料的外貌结构进行表征。如图1a所示，Fe_3_O_4_纳米粒子呈现均匀的球状形态，而当TpDT将Fe_3_O_4_纳米粒子交联在一起后，形成了不规则的颗粒状结构（图1b）。这表明TpDT已成功掺杂在Fe_3_O_4_纳米粒子表面。

**图1:**
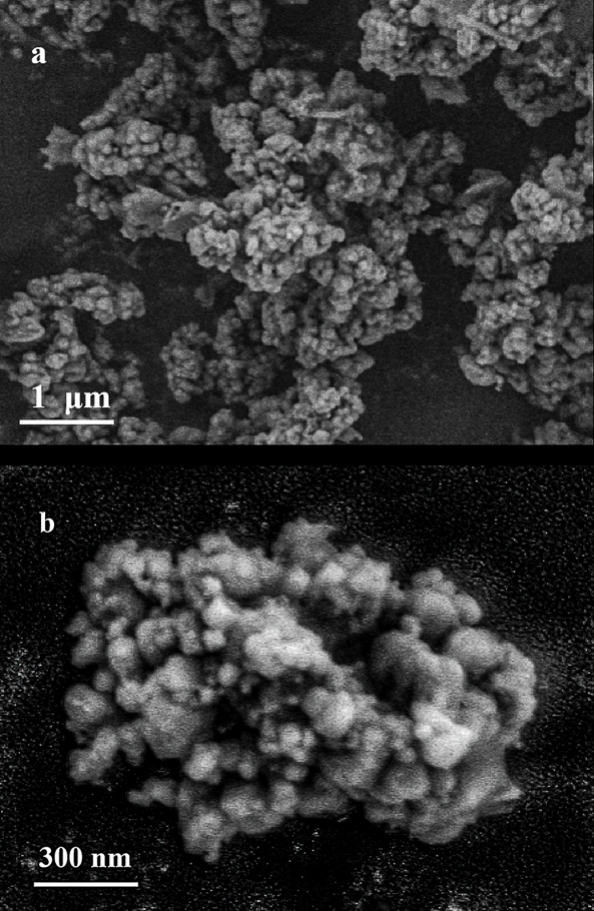
（a）Fe_3_O_4_和（b）Fe_3_O_4_@TpDT的SEM图

如图2所示，对比Fe_3_O_4_、TpDT和Fe_3_O_4_@TpDT的FT-IR图可知，Fe_3_O_4_@TpDT在1451 cm^‒1^和1572 cm^‒1^处出现的吸收峰归属于TpDT中C=C的伸缩振动峰^［27］^，Fe_3_O_4_@TpDT在1286 cm^‒1^和1616 cm^‒1^处出现的吸收峰归属于TpDT中C-N和C=O的伸缩振动峰^［26］^，Fe_3_O_4_@TpDT在589 cm^‒1^处出现的吸收峰来源于Fe_3_O_4_中的Fe-O-Fe^［20］^。上述实验结果与文献［^28^］报道一致，证明了Fe_3_O_4_@TpDT材料的成功合成。

**图2:**
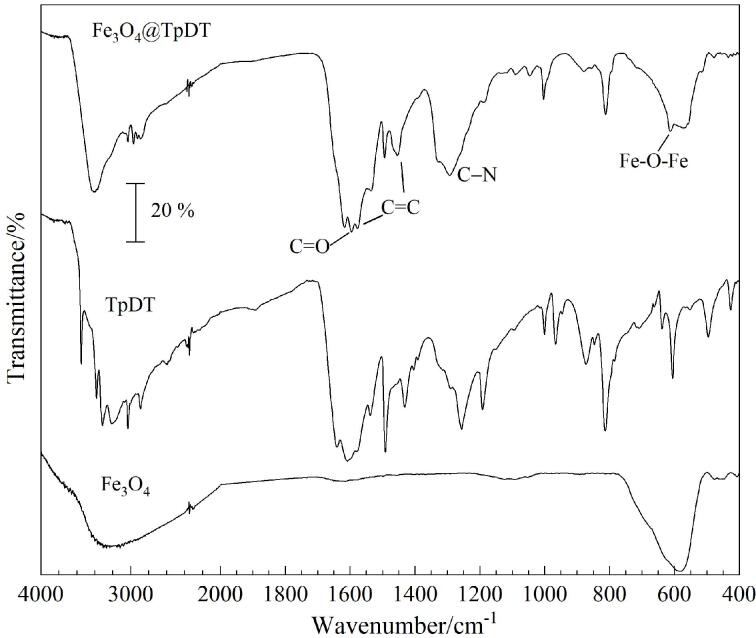
Fe_3_O_4_@TpDT、TpDT和Fe_3_O_4_的FT-IR图

利用XRD对Fe_3_O_4_@TpDT和Fe_3_O_4_的晶体结构进行表征，结果如图3所示。在10°~80°的2*θ*区域内，Fe_3_O_4_@TpDT中30.1°、35.5°、43.1°、53.5°、57.0°和62.6°处的衍射峰分别是（220）、（311）、（400）、（422）、（511）和（440），对应了Fe_3_O_4_的晶体结构，与文献［^28^］报道结果一致。Fe_3_O_4_@TpDT在18.4°和26.4°处出现了微弱的COF特征衍射峰，对应于（210）和（001）晶面，与文献［^27^］报道结果一致。上述XRD结果均证明了Fe_3_O_4_@TpDT的成功制备。

**图3:**
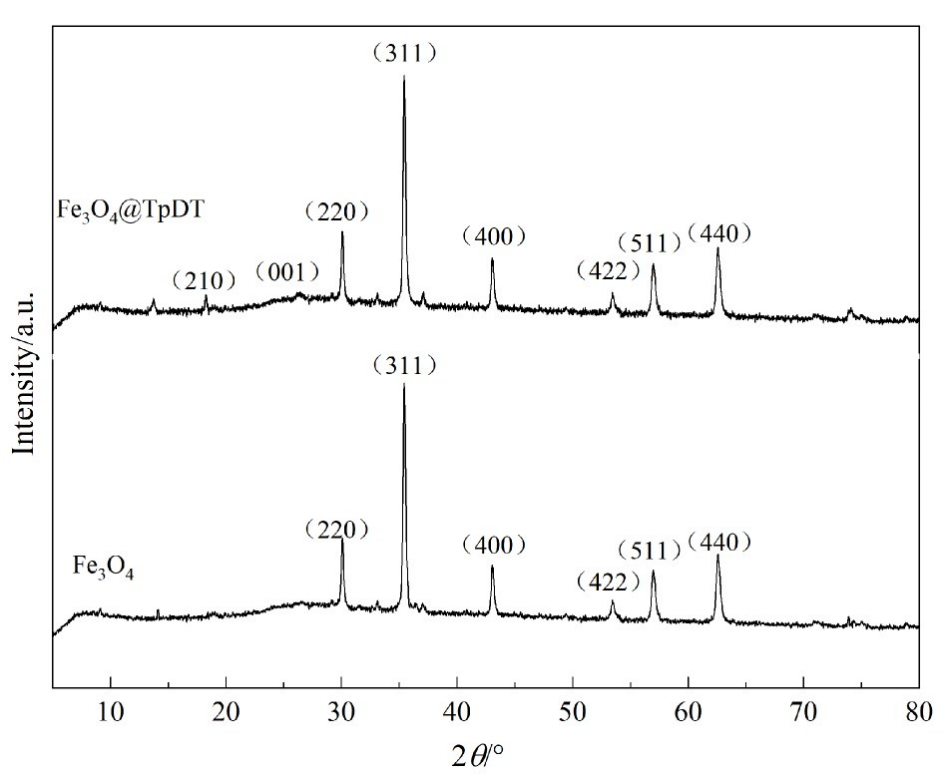
Fe_3_O_4_@TpDT和Fe_3_O_4_的XRD图

Fe_3_O_4_@TpDT材料的N_2_吸附-解吸等温线如图4所示，显示了Ⅳ型N_2_吸附等温线，展现了介孔结构特征。Fe_3_O_4_@TpDT的比表面积为81.83 m²/g，总孔体积为0.14 cm³/g，孔径为6.813 nm。基于密度泛函理论（density functional theory， DFT），运用Materials Studio软件中的Dmol³模块对目标化合物的结构进行优化并计算其分子尺寸。结果表明，8种SAs的分子尺寸为1.01~1.39 nm，当吸附材料的孔径比目标化合物的分子尺寸大3~6倍时，该吸附材料能够展现出良好的吸附与再生性能^［29］^。鉴于此，Fe_3_O_4_@TpDT的孔径适用于SAs的富集。

**图4:**
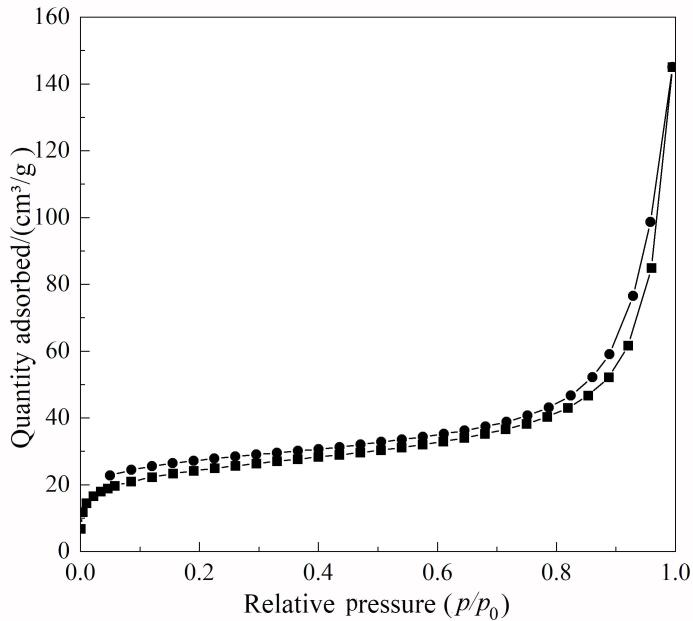
Fe_3_O_4_@TpDT的N_2_吸附-解吸等温线

采用XPS技术对Fe_3_O_4_@TpDT和TpDT的化学组成进行分析。如图5a所示，在Fe_3_O_4_@TpDT的C 1*s* XPS谱图中，于结合能284.8、286.3和291.2 eV处出现了3个峰，这些峰分别对应于TpDT中的C-C键、C-N键和*π*-*π*键^［30］^。如图5b所示，在Fe_3_O_4_@TpDT的O 1*s*谱图中，发现4个峰，其结合能分别位于530.3、531.2、532.8和533.5 eV处，这些峰分别归属于Fe-O、C-O、O-H和C=O^［31］^。Fe-O键的出现表明TpDT与Fe_3_O_4_实现了有效的复合。

**图5:**
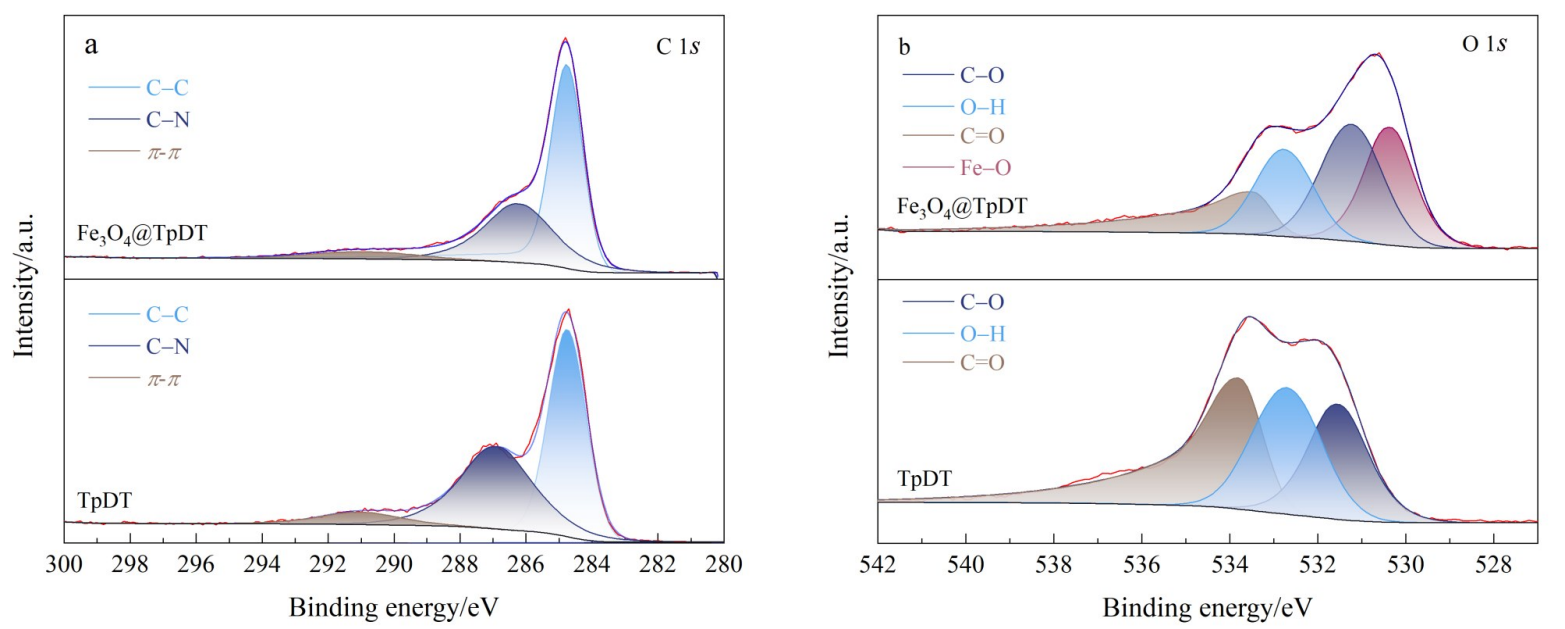
Fe_3_O_4_@TpDT和TpDT的（a）C 1*s*和（b）O 1*s* XPS谱图

### 2.2 色谱条件的优化

实验分别比较了0.1%甲酸水溶液-乙腈和0.1%甲酸水溶液-甲醇作为流动相时目标化合物的分离效果。结果表明，使用0.1%甲酸水溶液-甲醇流动相体系时，8种SAs的分离效果和峰形均较好，因此实验选择0.1%甲酸水溶液-甲醇作为流动相。8种SAs的提取离子色谱图见图6。

**图6:**
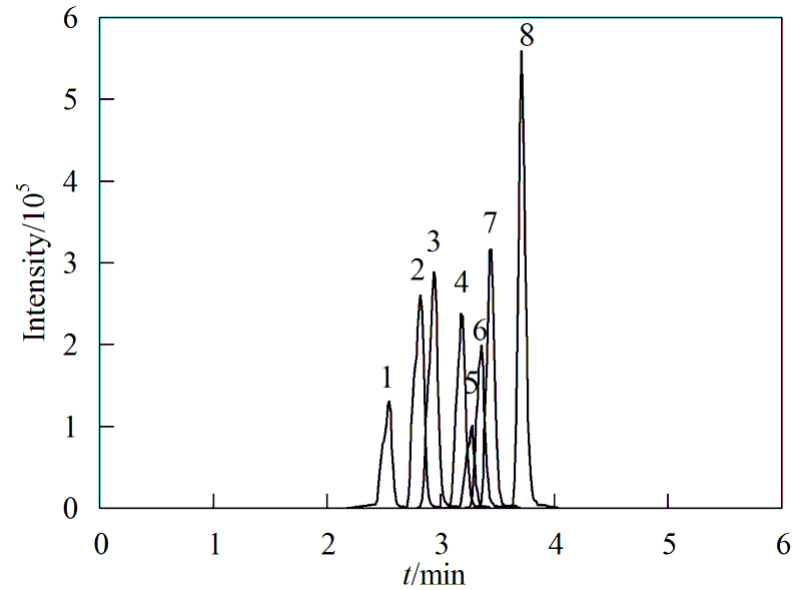
8种SAs的提取离子色谱图 Peak identifications： 1. SMI； 2. SMZ； 3. SMP； 4. SCP； 5. SMX； 6. SMM； 7 SIZ； 8. SB.

### 2.3 样品前处理条件的优化

为获得最佳萃取效率，对萃取条件（材料用量、水样pH、吸附时间）和洗脱条件（洗脱溶剂类型及体积、洗脱时间）进行优化考察。

#### 2.3.1 材料用量

实验考察了Fe_3_O_4_@TpDT用量分别为10、15、20、25和30 mg时对目标化合物的萃取效果。结果如图7a所示，当Fe_3_O_4_@TpDT的用量增加至25 mg时，萃取回收率达到最佳，继续增加用量，萃取回收率无明显变化，说明材料对目标化合物的吸附已接近饱和状态。因此，确定Fe_3_O_4_@TpDT磁性材料的用量为25 mg。

**图7:**
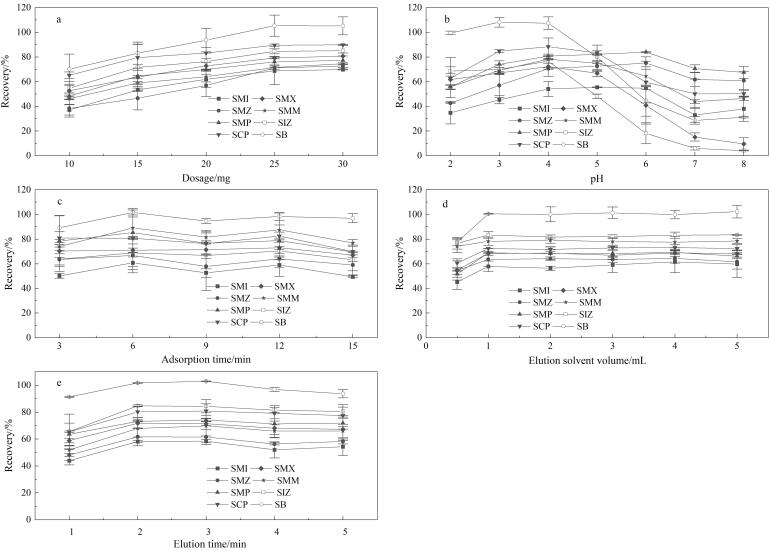
（a）Fe_3_O_4_@TpDT材料用量、（b）水样pH、（c）吸附时间、（d）洗脱溶剂体积和（e）洗脱时间对8种SAs回收率的影响（*n*=3）

#### 2.3.2 水样pH

样品溶液的pH是影响目标化合物回收率的重要因素之一，它与样品溶液中目标化合物的存在状态密切相关。SAs呈酸碱两性，其极弱的酸性源于磺酰胺基，对应的p*K*_a_为5~8，碱性则源于芳伯氨基^［1］^。实验分别考察了不同水样pH（2、3、4、5、6、7、8）对目标化合物回收率的影响，结果如图7b所示。当水样pH=4时，SAs以中性分子及两性离子的形态存在，这种存在形态有利于Fe_3_O_4_@TpDT通过*π*-*π*作用对SAs进行吸附，此时绝大部分SAs的回收率达到最高。随着水样pH逐渐增大，处于阴离子状态的SAs开始增多，这可能不利于SAs中的磺酰基与Fe_3_O_4_@TpDT中的N-H之间形成氢键^［18］^，进而导致多数SAs的回收率开始下降。因此，在实验中选择将水样的pH调节为4。

#### 2.3.3 吸附时间

考察了不同吸附时间（3、6、9、12、15 min）对SAs的吸附效果。如图7c所示，当吸附时间从3 min延长至6 min时，回收率有所提升，7种SAs的回收率达到最大；继续延长吸附时间，回收率并无明显提升或略有下降。因此，实验选定6 min作为最优吸附时间。

#### 2.3.4 洗脱条件

实验主要考察了洗脱溶剂类型及体积和洗脱时间对目标化合物回收率的影响。在MSPE过程中，洗脱溶剂类型是影响回收率的重要因素。实验选取3种溶剂（甲醇、乙腈和丙酮）作为SAs的洗脱溶剂，结果表明，甲醇的洗脱能力高于乙腈和丙酮。因此，选择甲醇作为本实验的洗脱溶剂。

考察了洗脱溶剂体积分别为0.5、1、2、3、4和5 mL时的单次洗脱条件下的洗脱效果。如图7d所示，当洗脱溶剂体积为1 mL时，8种SAs的回收率基本达到最大值，继续增大洗脱溶剂体积，回收率基本保持不变。因此，选择1 mL作为最佳洗脱溶剂体积。此外，将洗脱时间分别设置为1、2、3、4和5 min，以探究洗脱时间对8种SAs回收率的影响。如图7e所示，当洗脱时间为2 min时，洗脱溶剂对目标化合物的洗脱效果最佳，继续增加洗脱时间，目标化合物的回收率无明显变化或略有下降。因此，选择2 min作为最佳洗脱时间。

综上，本研究采取的最佳萃取条件为吸附剂用量25 mg，水样pH 4，吸附时间6 min，洗脱溶剂甲醇，洗脱溶剂体积1 mL，洗脱时间2 min。经计算，在最佳萃取条件下，Fe_3_O_4_@TpDT对SMI、SMZ、SMP、SCP、SMX、SMM、SIZ和SB的富集因子（EFs）分别为39、43、41、44、42、45、46和48。

### 2.4 基质效应

在使用UPLC-MS/MS进行定量分析时，基质效应（matrix effect，ME）会对分析结果的准确度产生影响。由于海水和水库水的成分较为复杂，因此需考虑基质效应所产生的影响。参照文献［^27^］方法，用水厂水、海水、水库水3类基质溶液和纯溶剂分别配制系列质量浓度（4、10、20、50、200、500、800、1 000 ng/L）的基质匹配混合标准溶液和溶剂混合标准溶液。根据ME=（基质匹配标准曲线斜率/溶剂标准曲线斜率‒1）×100%对基质效应进行评估。当|ME|<20%时，表示存在弱基质效应，无需采取补偿措施；当20%≤|ME|≤50%时，表示存在中等基质效应，需采取补偿措施；当|ME|>50%时，表示存在强基质效应，也需采取补偿措施^［32］^。结果如表2所示，除SMM、SMZ、SB、SMX和SIZ在水厂水中表现为弱基质效应以及SMX在水库水中表现为弱基质效应外，其余SAs在3种环境水样中均表现为中等基质效应。因此，为满足快速、准确的分析要求，水厂水、水库水和海水均采用基质匹配标准曲线进行定量分析。

**表2:** 8种SAs的基质效应

Analyte	MEs/%		
	Water treatment plant effluent	Reservoir water	Seawater
SMM	12.3	20.2	38.1
SMZ	18.4	26.3	44.0
SB	16.3	24.1	38.9
SMP	22.5	29.5	46.7
SMX	11.0	19.6	34.5
SCP	26.7	33.3	45.9
SMI	26.0	33.8	44.9
SIZ	16.0	24.4	39.3

### 2.5 方法学验证

#### 2.5.1 线性范围、检出限和定量限

用3种空白水样（水厂水、水库水、海水）基质液配制系列质量浓度（4、10、20、50、200、500、800、1 000 ng/L）的模拟水样，经样品前处理后上机检测，以质量浓度为横坐标（*x*， ng/L）、峰面积为纵坐标（*y*）绘制标准曲线。结果表明，SMM、SMZ、SB和SMP在4~1 000 ng/L范围内具有良好的线性关系，SMX、SCP和SMI在10~1 000 ng/L范围内具有良好的线性关系，SIZ在20~1 000 ng/L范围内具有良好的线性关系，相关系数（*r* ^2^）均≥0.992 6，8种SAs的检出限（LOD，*S/N*=3）和定量限（LOQ，*S/N*=10）分别为0.80~3.44 ng/L和2.66~11.47 ng/L。以水厂水基质为例，相关数据见表3。

**表3:** 8种SAs的线性范围、线性方程、相关系数、检出限和定量限^*^

Analyte	Linear range/（ng/L）	Linear equation	*r* ^2^	LOD/（ng/L）	LOQ/（ng/L）
SMM	4‒1000	*y*=52.05*x‒*365.80	0.9973	0.80	2.66
SMZ	4‒1000	*y*=93.70*x‒*1259.30	0.9926	0.89	2.97
SB	4‒1000	*y*=135.74*x‒*2434.20	0.9932	1.11	3.71
SMP	4‒1000	*y*=75.41*x‒*533.62	0.9972	1.15	3.82
SMX	10‒1000	*y*=31.90*x‒*255.08	0.9933	1.60	5.34
SCP	10‒1000	*y*=70.46*x‒*728.71	0.9967	1.65	5.49
SMI	10‒1000	*y*=43.77*x‒*742.15	0.9926	2.68	8.95
SIZ	20‒1000	*y*=69.10*x‒*1057.20	0.9946	3.44	11.47

* water treatment plant effluent. *y*： peak area； *x*： mass concentration， ng/L.

#### 2.5.2 回收率和精密度

在空白水样（水厂水）中添加低、中、高3个水平（50、500和800 ng/L）的混合标准工作溶液，进行加标回收试验，计算加标回收率；每个加标水平1 d内测定6个平行样，计算日内精密度；连续测定6 d，计算日间精密度，结果见表4。

**表4:** 8种SAs的加标回收率、日内和日间精密度（*n*=6）

Analyte	Spiked level/（ng/L）	Recovery/%	Intra-day RSD/%	Inter-day RSD/%
SMM	50	87.1	4.6	8.7
	500	85.8	7.7	9.9
	800	90.5	8.6	11.3
SMZ	50	86.6	9.0	10.3
	500	78.1	9.1	17.8
	800	73.0	8.9	15.4
SB	50	79.3	6.0	8.9
	500	103.5	8.2	9.4
	800	112.9	7.9	8.7
SMP	50	80.3	10.4	13.9
	500	84.9	6.9	15.9
	800	82.0	12.5	17.0
SMX	50	80.3	8.7	12.5
	500	85.0	11.7	9.8
	800	88.6	5.6	17.4
SCP	50	86.0	7.6	13.2
	500	95.7	8.1	17.2
	800	95.6	6.7	13.9
SMI	50	76.0	6.5	11.2
	500	74.8	8.6	9.8
	800	75.1	4.4	8.7
SIZ	50	93.9	9.4	11.2
	500	82.0	8.5	12.3
	800	87.7	8.8	19.2

由表4可以看出，8种SAs的加标回收率为73.0%~112.9%，日内和日间精密度分别为4.4%~12.5%和8.7%~19.2%。实验结果表明，所建方法具有良好的精密度，能够用于水样中8种SAs的测定。

### 2.6 实际样品分析

将所建方法用于水厂水、水库水和海水中SAs的测定。结果如表5所示，水厂水中均未检出8种SAs；水库水中检出3种SAs（SMM、SMX、SMI），含量为8.5~21.3 ng/L；海水中检出1种SAs（SMX），含量为11.8 ng/L。在水库水和海水中进行低、中、高3个水平（50、500和800 ng/L）的加标回收率试验，每个加标水平测定3个平行样，8种SAs的加标回收率为55.0%~100.9%，相对标准偏差（RSD）为1.3%~14.0%（相关数据结果见附表1），结果表明该方法适用于环境水样中SAs的富集和检测。

**表5:** 实际水样中8种SAs的测定结果（*n*=3）

Analyte	Water treatment plant effluent/（ng/L）	Reservoir water/（ng/L）	Seawater/（ng/L）
SMM	ND	8.5	ND
SMZ	ND	ND	ND
SB	ND	ND	ND
SMP	ND	ND	ND
SMX	ND	15.9	11.8
SCP	ND	ND	ND
SMI	ND	21.3	ND
SIZ	ND	ND	ND

ND： not detected.

### 2.7 Fe_3_O_4_@TpDT吸附材料与HLB柱的比较

HLB固相萃取柱是目前在水样中萃取SAs时最常用的商品化材料，其萃取基质由*N*-乙烯吡咯烷酮和二乙烯基苯按特定比例聚合而成。分别按照GB/T 5750.8-2023^［33］^中关于药品及个人护理品的萃取方法以及1.4节前处理方法，使用HLB固相萃取柱和Fe_3_O_4_@TpDT吸附材料，对添加了SMI、SMX、SMZ、SCP（加标水平均为500 ng/L）的空白水厂水样品进行萃取效果的比较，比较指标包括吸附时间、洗脱时间和回收率。结果如表6所示，HLB固相萃取柱与Fe_3_O_4_@TpDT材料对4种SAs的加标回收率相当，但使用Fe_3_O_4_@TpDT材料所需要的吸附时间和洗脱时间均短于HLB柱。

**表6:** Fe_3_O_4_@TpDT吸附材料与HLB固相萃取柱的萃取效果

Material	Analyte	Adsorption time/min	Elution time/min	Recovery/%
HLB SPE column	SMI	18	12	69.4
	SMX	18	12	84.5
	SMZ	18	12	83.3
	SCP	18	12	78.4
Fe_3_O_4_@TpDT	SMI	6	2	74.3
	SMX	6	2	86.2
	SMZ	6	2	77.4
	SCP	6	2	88.5

### 2.8 与文献方法的比较

将所建方法与其他环境水样中SAs的检测方法进行比较（表7）。可以看出，采用本方法仅需6 min即可完成SAs的吸附过程，与其他文献方法相比，本方法的萃取时间更短。此外，结合UPLC-MS/MS进行检测，本方法获得了较低的LOD。

**表7:** 本方法与文献方法的比较

Material	Detection method	Matrices	Extraction time/min	LODs	Ref.
DMIPs	DSPE-HPLC-UV	lake water and seawater	35	0.27‒0.64 μg/L	［^34^］
ILs@Zr-MOFs	DSPE-HPLC-DAD	environmental water	10	0.01‒0.03 μg/L	［^35^］
TPB-DMTP-COFs	SPE-HPLC-MS/MS	spring water and pond water	>30	0.5‒1.0 ng/L	［^36^］
Fe_3_O_4_@ZIF-8	MSPE-HPLC-MS/MS	drinking water and river water	15	0.06‒0.71 μg/L	［^17^］
HLB	SPE-HPLC-MS/MS	environmental water	>160	0.1‒0.5 μg/L	［^14^］
MIPs	MSPE-HPLC-MS/MS	environmental water	10	1.4‒2.8 ng/L	［^37^］
IL@MGO	MSPE-UPLC-MS/MS	environmental water	12	0.75‒1.47 ng/L	［^38^］
Fe_3_O_4_@TpDT	MSPE-UPLC-MS/MS	water treatment plant effluent， reservoir water and seawater	6	0.8‒3.4 ng/L	this work

DMIPs： dummy template molecularly imprinted polymers； ILs： imidazole-based ionic liquids； MOFs： metal organic frameworks； COFs： covalent organic frameworks； TPB： triphenylbenzene； DMTP： dimethoxyterephthaldehyde； ZIF： zeolitic imidazolate frameworks； MIPs： molecularly imprinted polymers； MGO： magnetic graphene oxide.

## 3 结论

本文首先制备了磁性COF材料Fe_3_O_4_@TpDT，将其作为MSPE吸附材料，结合UPLC-MS/MS技术，建立了一种测定环境水样中8种SAs的分析方法。该方法具备较高的灵敏度、精密度和准确性，且样品前处理耗时较短，能够为环境水样中SAs的残留分析提供有力的技术支持。
